# IL‐6 Promotes Pathological Angiogenesis in Oral Submucous Fibrosis by Suppressing USP10–VEGFR1 Interaction

**DOI:** 10.1111/jcmm.71324

**Published:** 2026-08-19

**Authors:** Chunyu Li, Lingshuang Han, Zhenhao Wang, Yihang Xie, Xiaoping Xu, Qianming Chen, Liang Xie

**Affiliations:** ^1^ State Key Laboratory of Oral Diseases, National Center for Stomatology, National Clinical Research Center for Oral Diseases, Research Unit of Oral Carcinogenesis and Management, Chinese Academy of Medical Sciences West China Hospital of Stomatology, Sichuan University Chengdu Sichuan China; ^2^ Stomatology Hospital, School of Stomatology, Zhejiang University School of Medicine, Zhejiang Provincial Clinical Research Center for Oral Diseases, Key Laboratory of Oral Biomedical Research of Zhejiang Province, Engineering Research Center of Oral Biomaterials and Devices of Zhejiang Province Hangzhou China; ^3^ The Central Laboratory Peking University School and Hospital of Stomatology Beijing China

**Keywords:** angiogenesis, IL‐6, oral mucosa, oral submucous fibrosis, USP10

## Abstract

Oral submucous fibrosis (OSF) is a chronic, progressive, premalignant disorder with expanding epidemiology, rising annual incidence, and increasing malignant transformation rates. Therefore, elucidating its pathogenesis and establishing early intervention strategies remain urgent priorities. In this study, we evaluated the interplay between USP10 and IL‐6 and its potential influence on the development of oral submucous fibrosis. Immunohistochemistry and immunofluorescence were used to examine the correlation among IL‐6, USP10, and pathological angiogenesis in OSF clinical samples and mouse models, revealing high IL‐6 expression in the inflammatory microvasculature alongside downregulated USP10. Functional experiments on human umbilical vein endothelial cells (HUVECs) in vitro, including administration of recombinant human IL‐6, demonstrated that USP10 inhibited angiogenesis, cell migration, and cell proliferation, and that targeting USP10 reversed IL‐6‐mediated pathological angiogenesis. RNA sequencing further identified differentially expressed genes in HUVECs, while mass spectrometry, western blotting, and co‐immunoprecipitation uncovered that USP10 interacts with vascular endothelial growth factor receptor 1 (VEGFR1) and inhibits its ubiquitination, thereby suppressing angiogenesis. Collectively, these findings indicate that IL‐6 promotes pathological angiogenesis by suppressing USP10 and its interaction with VEGFR1, thus accelerating OSF progression, and suggest that targeting USP10 may represent a promising mechanism for further investigation in OSF therapy.

## 1 Introduction

Oral submucous fibrosis (OSF) is a chronic, progressive, and potentially malignant disorder. It is characterized by progressive fibrotic changes in the submucosal tissues of the oral cavity and oropharynx, primarily associated with areca nut chewing. Clinically, OSF manifests as fibrous bands in the buccal mucosa, lip mucosa, and palate mucosa, leading to progressive mouth opening limitation. With a global prevalence rate of 4.96%, OSF exhibits distinct geographical clustering, particularly in regions such as Hunan, Hainan and other places in China. The malignant transformation rate of untreated OSF to oral squamous cell carcinoma (OSCC) ranges from 7% to 13% over varying time periods [1, 2, 3]. As the epidemiological scope of OSF continues to expand, the number of affected individuals is increasing annually, and the malignant transformation rate is rising significantly, which seriously damages the health of patients and affects their quality of life. Consequently, elucidating the pathogenesis of OSF and developing effective strategies for early intervention is one of the urgent problems in both clinical practice and scientific research.

Early pathological changes in OSF include mild vascular hyperplasia, inflammatory cell infiltration, increased collagen fibre deposition, scattered fibroblast distribution, collagen homogenization, and hyalinization near the epithelium [1, 2, 3, 4]. However, the resulting vessels are immature and fail to alleviate tissue hypoxia. Instead, they contribute to increased cytokine production, further extracellular matrix accumulation, and accelerated fibrotic progression of OSF [5]. Currently, anti‐inflammatory drugs such as glucocorticoids have been widely employed in clinical management of OSF, demonstrating efficacy in improving symptoms and clinical signs of patients [6]. In summary, pathological angiogenesis induced by vascular inflammation plays a pivotal role in the occurrence and development of OSF. Nevertheless, the mechanisms by which vascular inflammation drives pathological angiogenesis in OSF and the molecular regulatory networks involved remain poorly understood. Therefore, elucidating the specific role of pathological angiogenesis in OSF progression and its molecular mechanisms may facilitate the development of new strategies for early diagnosis and intervention.

The early stages of OSF are characterized by a localized inflammatory response involving the secretion of various inflammatory mediators, including interleukin‐6 (IL‐6), interleukin‐8 (IL‐8), interleukin‐1β (IL‐1β), tumour necrosis factor‐α (TNF‐α), and transforming growth factor‐β (TGF‐β) [3]. IL‐6, a pivotal pro‐inflammatory cytokine, is secreted by lymphocytes, fibroblasts, endothelial cells, and other cell types. It plays a critical role in regulating inflammatory responses, haematopoiesis, and immune processes [7, 8]. Recent studies have demonstrated that IL‐6 can enhance the angiogenic properties of microglia, thereby promoting cerebral vascular repair and functional recovery [9]. Chih‐Yu Peng et al. employed RNA sequencing to demonstrate upregulation of IL‐6 expression in OSF tissues relative to normal tissues [10]. In vitro studies further corroborated these findings, showing that IL‐6 expression in fibroblasts from OSF patients was markedly higher than in those from healthy controls. Additionally, the expression of IL‐6 in fibroblasts exhibited a dose‐dependent increase following treatment with arecoline [11]. Collectively, these findings underscore the significant role of IL‐6 in angiogenesis and inflammatory responses, suggesting its potential involvement in the occurrence and progression of OSF. Nevertheless, the precise mechanistic role of IL‐6 in OSF remains to be elucidated.

Ubiquitination is a post‐translational modification process in which ubiquitin molecules are covalently attached to target proteins through the action of a cascade of enzymatic reactions. This process plays a critical role in numerous physiological mechanisms, including autophagy, DNA damage repair, cell proliferation, and cell apoptosis. Deubiquitination refers to the removal of ubiquitin molecules from ubiquitinated substrate proteins, mediated by deubiquitinating enzymes [12]. Through post‐translational modification of various substrate proteins, ubiquitination regulates the balance between pro‐angiogenic and anti‐angiogenic factors, ensuring precise control over complex processes such as angiogenesis. Key pathways influenced by ubiquitination include hypoxia‐induced angiogenesis, vascular endothelial growth factor receptor (VEGFR) signalling, and the NOTCH pathway, all of which contribute to the regulation of angiogenesis [13]. Angiogenesis is a physiological and pathological process regulated by multiple factor families. Among these, VEGFA and its receptors VEGFR1 and VEGFR2 constitute the central signalling axis governing angiogenic responses [14]. VEGFR1 is expressed on endothelial and haematopoietic cells and functions as a negative regulator of VEGFA signalling. With a higher affinity for VEGFA than VEGFR2, VEGFR1 can competitively bind VEGFA, thereby negatively regulating angiogenesis [14, 15, 16]. Fong et al. reported that embryonic lethality occurred in VEGFR1 knockout mice, attributed to excessive endothelial cell proliferation and concomitant abnormal angiogenesis [17]. In zebrafish, Krueger et al. demonstrated that knockdown of VEGFR1 can promote angiogenesis [18]. Stefater et al. showed that WNT signalling–induced expression of VEGFR1 in retinal myeloid cells inhibits angiogenesis in the mouse retina [19]. Ubiquitin‐specific peptidase 10 (USP10), a member of the deubiquitinating enzyme family, is extensively expressed in human tissues, and its dysregulation has been implicated in various diseases. Predominantly localized in the cytoplasm, USP10 modulates critical cellular processes such as the cell cycle, cell proliferation, and cell apoptosis through its deubiquitination activity [20]. In 2019, a study revealed that USP10 can reverse ubiquitination of Notch1 by interacting with NICD1, the intracellular domain of Notch1. This interaction increases the abundance and stability of the Notch1 in vascular endothelial cells under nutrient or hypoxic conditions, ultimately inhibiting endothelial cell sprouting and vascularization [21]. While this finding demonstrates the involvement of USP10 in angiogenesis, its role in OSF remains unexplored.

In this study, using clinical samples, animal models, cell experiments, and transcriptome analysis, we clarified the influence of inflammatory factors on angiogenesis by regulating USP10 during the initiation and progression of OSF. We explored the core components and mechanisms by which USP10 regulates angiogenesis and analysed the potential to delay or reverse OSF progression by targeting USP10. This study provides preliminary observations that may inform future studies of clinical OSF.

## 2 Materials and Methods

### 2.1 Patient Tissue Samples

Normal and early stage of OSF biopsy tissues were obtained from the West China Hospital of Stomatology. The pathological criteria for diagnosing early stage of OSF are the same as those mentioned in the preceding introduction. Normal human tissues were procured from healthy oral mucosa during orthognathic surgery or wisdom tooth extraction. Early stage of OSF tissues were collected from bioptic OSF lesions without any prior treatment. Table S1 shows clinicopathological features of patients with OSF. Prior to the study, written informed consent was obtained from all participants. The study was approved by the Ethics Committee of the West China Hospital of Stomatology, Sichuan University (WCHSIRB‐D‐2023‐267).

### 2.2 Immunohistochemistry (IHC)

The tissue samples were fixed in 4% paraformaldehyde, embedded in paraffin, and subsequently dewaxed using xylene. Following hydration with anhydrous ethanol, antigen retrieval was performed. The samples were then blocked with 3% hydrogen peroxide solution for 15 min and 5% bovine serum albumin (BSA) for 30 min. Primary antibodies, including IL‐6 antibody (AFFINITY, DF6087, 1:200) and USP10 antibody (Abcam, ab70895, 1:200), were applied to the tissues and incubated overnight at 4°C. On the following day, the tissues were treated with goat anti‐rabbit secondary antibody (ZSGB‐BIO, PV6001) and incubated at 37°C for 30 min. Colour development was achieved using 2,3‐diaminobenzidine (DAB) kit (ZSGB‐BIO, ZLI9018), with Solution A and Solution B mixed at a 1:100 ratio. The reaction was terminated by rinsing with tap water, followed by counterstaining with haematoxylin. The slides were washed under running water to achieve blueing, air‐dried, and mounted with neutral gum. We used German semi‐quantitative scoring system evaluates both staining intensity and area extent. The percentage of staining was given a score of 0 (< 5%), 1 (5%−25%), 2 (25%–50%), 3 (50%−75%), or 4 (> 75%). Besides, staining intensity was defined as: 0, no staining; 1, weak staining; 2, moderate staining; and 3, strong staining. These two scores were multiplied as the final score.

### 2.3 Immunofluorescence(IF)

The tissue samples were fixed in 4% paraformaldehyde, embedded in paraffin, and subsequently dewaxed using xylene. Following hydration with anhydrous ethanol, antigen retrieval was performed. The tissues were then blocked with 5% BSA for 30 min. Primary antibodies, including USP10 (Abcam, ab70895, 1:200) and CD31 (Abcam, ab9498, 1:200), were applied to the tissue sections. After overnight incubation at 4°C, the sections were treated with fluorescent secondary antibodies: goat anti‐rabbit (ZSGB‐BIO, ZF‐0311, 1:200) and goat anti‐mouse (ZSGB‐BIO, ZF‐0313, 1:200), followed by incubation at 37°C for 90 min.

The cell nuclei were stained with Antifade Mounting Medium with DAPI (Vector Laboratories, H‐1200). The IF staining intensity was semi‐quantified using ImageJ. Conduct the following analysis in the software: split channels–adjust threshold–measure. Mean density = Integrated density/Area.

All IHC and IF slides were assessed and confirmed independently by two experienced pathologists who were blinded to the experimental groups. Interobserver agreement was assessed using Cohen's kappa coefficient, indicating excellent agreement. Discrepancies were resolved by consensus discussion.

### 2.4 OSF Mouse Model

Forty 6‐week‐old male Balb/c mice were obtained from Chengdu Dossy Experimental Animals Co. Ltd. The mice were randomly allocated into three groups: the experimental group, the phosphate‐buffered saline (PBS) treatment group, and the sham operation group. In the experimental group, the left buccal mucosa of each mouse was injected daily with 60 μL of arecoline solution (20 μg/mL), while the right buccal mucosa served as the self‐control. In the PBS treatment group, the left buccal mucosa was injected daily with 60 μL of PBS solution. In the sham operation group, the left buccal mucosa was subjected to needle punching without any liquid injection, performed once daily. Buccal mucosa tissues were received at 8 and 12 weeks post‐administration, followed by fixation, dehydration, and embedding to prepare tissue sections.

Prior to the formal experiment, five arecoline concentrations were tested: 5, 10, 20, 50 and 100 μg/mL, with daily injections of 60 μL for 8 weeks.

The pre‐experiment results indicated that no fibrosis occurred in the mice of the 5 and 10 μg/mL groups whereas the 20, 50 and 100 μg/mL groups exhibited similar levels of fibrosis. Considering the toxicity of arecoline, the formal in vivo study employed 20 μg/mL.

### 2.5 Haematoxylin–Eosin Staining (HE Staining)

The paraffin sections were deparaffinized and rehydrated through a graded alcohol series. Subsequently, the sections were stained with haematoxylin for 50 s. The sections were then rinsed under running water for 10 min and counterstained with 0.5% eosin solution for 40 s.

### 2.6 Masson's Trichrome Staining

The paraffin sections were deparaffinized and rehydrated through an alcohol gradient. Subsequently, the sections were stained using a Masson's Trichrome Stain Kit (D026‐1‐1, NJJCBIO, Nanjing, China). The staining procedure involved the following steps: staining with Ponceau dye for 6 min; staining with phosphomolybdic acid solution for 5 min; staining with aniline blue solution for 2 min; staining with glacial acetic acid solution for 1 min.

### 2.7 Cell Culture

The HUVEC line was obtained from the American Type Culture Collection (ATCC). Cells were cultured in Dulbecco's Modified Eagle Medium (DMEM; Sigma‐Aldrich, St. Louis, MO, USA) supplemented with 10% fetal bovine serum (FBS; Thermo Fisher Scientific, Waltham, MA, USA) and 1% penicillin–streptomycin (Thermo Fisher Scientific) at 37°C with 5% CO_2_.

### 2.8 Stable Cell Line Generation

The lentiviruses used to infect HUVECs were obtained from Shanghai Genechem Co. Ltd. These included lentiviruses packaged with the USP10 overexpression plasmid, the corresponding negative control plasmid, the USP10 knockdown plasmid, and its respective negative control plasmid. To establish stable cell lines, the infected cells were cultured in a selective medium containing puromycin.

### 2.9 RNA Extraction and Quantitative Polymerase Chain Reaction (qPCR)

Total RNA was extracted from the cells using TRIzol reagent (Invitrogen, Carlsbad, CA, USA). Complementary DNA (cDNA) was synthesized from the isolated RNA using the RT reagent Kit with gDNA Eraser (Takara, Kusatsu, Japan). The sequences of the primers used for qPCR are listed below: USP10 forward: 5′‐GAGGGCACAGCTACCAACG‐3′, USP10 reverse: 5′‐AGGGGAGATAT GGCGGGAG‐3′; VEGFR1 forward: 5′‐TTTGCCTGAAATGGTGAGTAAGG‐3′, VEGFR1 reverse: 5′‐TGGTTTGCTTGAGCTGTGTTC‐3′; VEGFR2 forward: 5′‐CGGACAGTGGTATGGTTCTTGC‐3′, VEGFR2 reverse: 5′‐GTGGTGTCTGTGTCATCGGAGTG‐3′; ADM forward: 5′‐TCCCCCTATTTTAAGACGTGAATG‐3′, ADM reverse: 5′‐CATGCACACAAACACACTCACAT‐3′; DLL4 forward: 5′‐GTCTCCACGCCGGTATTGG‐3′, DLL4 reverse: 5′‐CAGGTGAAATTGAAGGGCAGT‐3′; GAPDH forward: 5′‐GGAGCGAGATCCCTCCAAAAT‐3′, GAPDH reverse: 5′‐GGCTGTTGTCATACTTCTCATGG‐3′. QPCR was performed using the SYBR Green system (Takara, Kusatsu, Japan) and run in an ABI 7300 Real‐time PCR instrument.

### 2.10 Western Blot Assay (WB Assay)

Total proteins were extracted using RIPA lysis buffer (Solarbio, Beijing, China) supplemented with a protease inhibitor cocktail (Solarbio). Protein samples (30 μg per lane) were separated by 10% sodium dodecyl sulfate‐polyacrylamide gel electrophoresis (SDS‐PAGE; Bio‐Rad, USA) and subsequently transferred onto 0.45 μm polyvinylidene fluoride (PVDF) membranes (Solarbio). The membranes were blocked with 5% skimmed milk at room temperature for 1 h and then incubated with primary antibodies at 4°C overnight. Following primary antibody incubation, the membranes were probed with horseradish peroxidase (HRP)‐conjugated secondary antibodies (ZSGB‐BIO, Beijing, China) at room temperature for 1 h.

The primary antibodies used in this study were as follows: USP10 (Abcam, ab70895, 1:1000), VEGFR1 (Abcam, ab2350, 1:1000), VEGFR2 (Proteintech, 26415, 1:1000), Ubiquitin (Santa Cruz, sc8017, 1:600) and β‐actin (ZSGB‐BIO, 700068, 1:10000).

### 2.11 Tube Formation Assay

A volume of 200 μL Matrigel was added to each well of a 48‐well plate and incubated at 37°C for 1 h. Subsequently, HUVECs (100 μL, 30,000 cells per well) were seeded onto the Matrigel‐coated wells and cultured for 8 h. The formation of tubular structures was observed and photographed under a microscope, and the images were quantitatively analysed using ImageJ software.

### 2.12 Cell Proliferation Assay

HUVECs (200 μL, 2000 cells per well) were seeded into a 96‐well plate. After cell adhesion, 10 μL of Cell Counting Kit‐8 (CCK‐8; Dojindo, Japan) solution was added to each well at 0, 24, 48, and 72 h, followed by incubation for 2 h at 37°C. The absorbance of each sample was measured at a wavelength of 450 nm.

### 2.13 Wound Healing Assay

Cells were seeded at a density of 2 × 10^5^ cells per well in 6‐well plates and incubated until reaching 90% confluent monolayer. The cell monolayer was scraped in a straight line with a 200 μL pipette tip to create a “scratch”. After washing the wells three times with PBS, images of the wound area were captured at the same position immediately after scratching (0 h) and at 6 h, 12 h, and 24 h post‐scratching. Cell migration (%) = migrated cell surface area/total surface area × 100%.

### 2.14 Differentially Expressed Genes Analysis

Transcriptome sequencing and analysis were performed by OE Biotech Co. Ltd. (Shanghai, China). The sequencing processes of database construction are as follows: total RNA extraction from cells, RNA quality detection, RNA fragmentation, reverse transcription to generate cDNA, adapter ligation, PCR amplification, Illumina HiSeq X 10 platform sequencing. After quantitative analysis of the gene expression level of each sample, DESeq2 software (1.16.1) was used to analyse the differential expression gene. The threshold for the significantly differential expression was *p* value < 0.05 and |log2foldchange (FC) | > 2.

### 2.15 Co‐Immunoprecipitation (Co‐IP) Assay

Cells (1 × 10^7^) were washed twice with pre‐cooled PBS. Subsequently, 400 μL of ice‐cold NP‐40 lysis buffer (Beyotime, P0013F) was added, and the cells were lysed for 30 min at 4°C. The lysate was centrifuged at 1200 × g for 10 min to collect the supernatant. To remove non‐specific binding, the supernatant was pre‐cleared using Protein G agarose beads (Santa Cruz, sc‐2003). The pre‐cleared supernatant was then incubated with anti‐USP10 antibody (Abcam, ab70895) and anti‐IgG antibody (Proteintech, 30000) on a rotary shaker overnight at 4°C. The following day, pre‐washed Protein G agarose beads were added to the mixture and incubated for 2 h. The beads were washed five times with diluted lysis buffer. Electrophoresis, membrane transfer, and immunoblotting were performed following standard western blotting protocols.

### 2.16 Statistical Analysis

All experiments were repeated at least three times in this manuscript. We analysed differences between groups by using a Student's *t*‐test and a one‐way ANOVA. Statistical analysis was performed using GraphPad Prism 9.0 software. *p* < 0.05 is considered significantly different (*p < 0.05, **p < 0.01, ***p < 0.001, ****p < 0.0001).

## 3 Results

### 3.1 IL‐6 Levels Are Elevated While USP10 Levels Are Reduced in the Vessels of Human OSF

**FIGURE 1:**
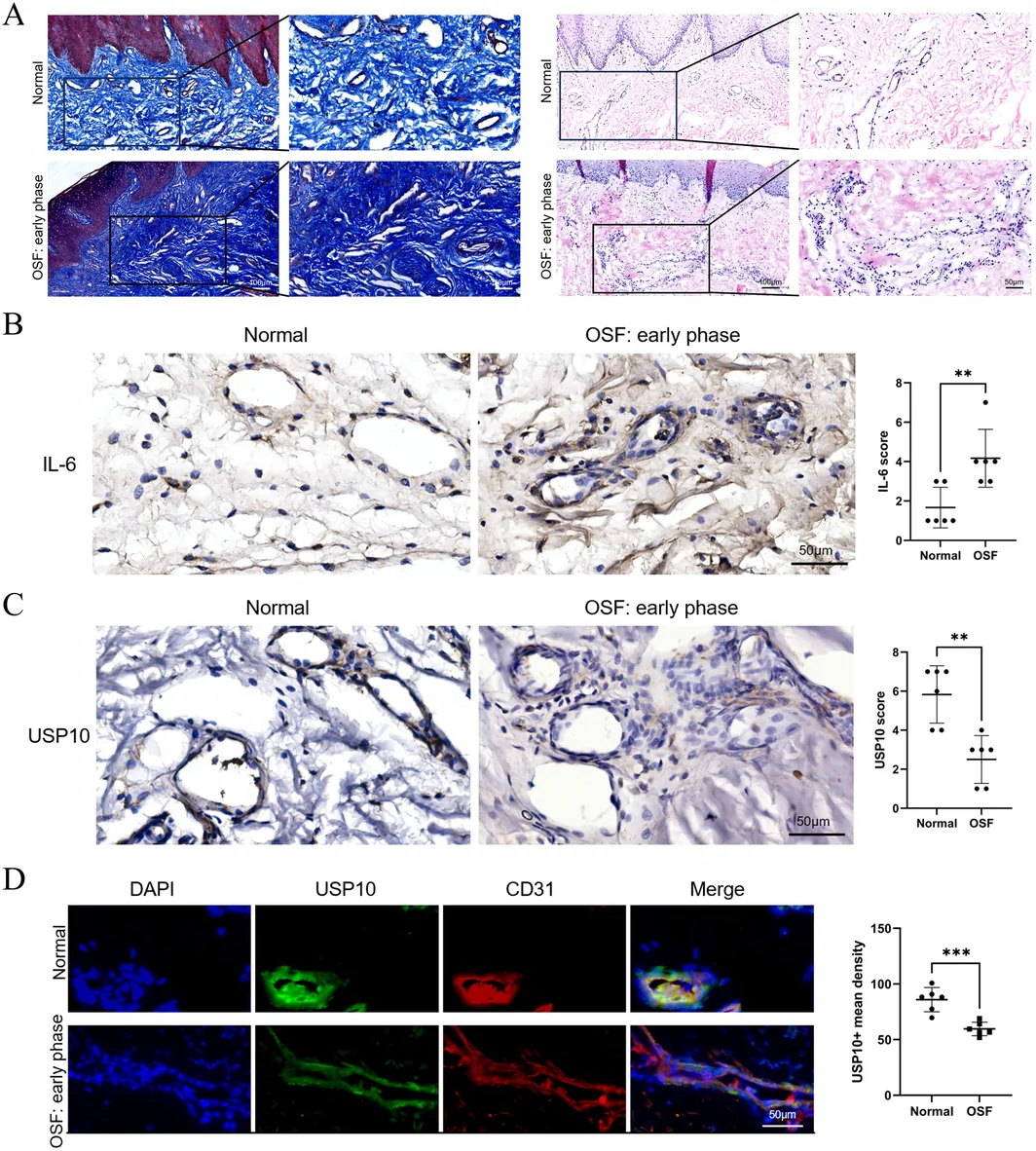
Upregulation of IL‐6 and downregulation of USP10 in human OSF (*n* = 6). (A) HE staining and Masson's trichrome staining in oral human samples, scale bar: Left panels, 100 μm; right panels, 50 μm. (B) Representative IHC images and analysis of IL‐6 in oral human samples, scale bar 50 μm. (C) Representative IHC images and analysis of USP10 in oral human samples, scale bar 50 μm. (D) Immunofluorescence and analysis of USP10 and CD31 in OSF, scale bar 50 μm. **p < 0.01, ***p < 0.001 by unpaired Student's *t* test.

In human tissues, we conducted HE staining and MASSON staining. The results revealed a higher density of blood vessels in early stage of OSF compared to normal controls, accompanied by a large number of inflammatory cells infiltrating the blood vessels and their surroundings, around which collagen fibres could be seen encircling them (Figure 1A). To explore the relationship between IL‐6, USP10 and inflammatory blood vessels in early‐stage OSF, we performed IHC and IF. The results indicated that IL‐6 expression was markedly elevated in inflamed blood vessels compared to normal oral tissues, whereas USP10 expression was significantly reduced. Notably, USP10 expression co‐localized with CD31 in vascular endothelial cells (Figure 1B–D).

### 3.2 IL‐6 Levels Are Elevated While USP10 Levels Are Reduced in the Vessels of Mouse OSF

**FIGURE 2:**
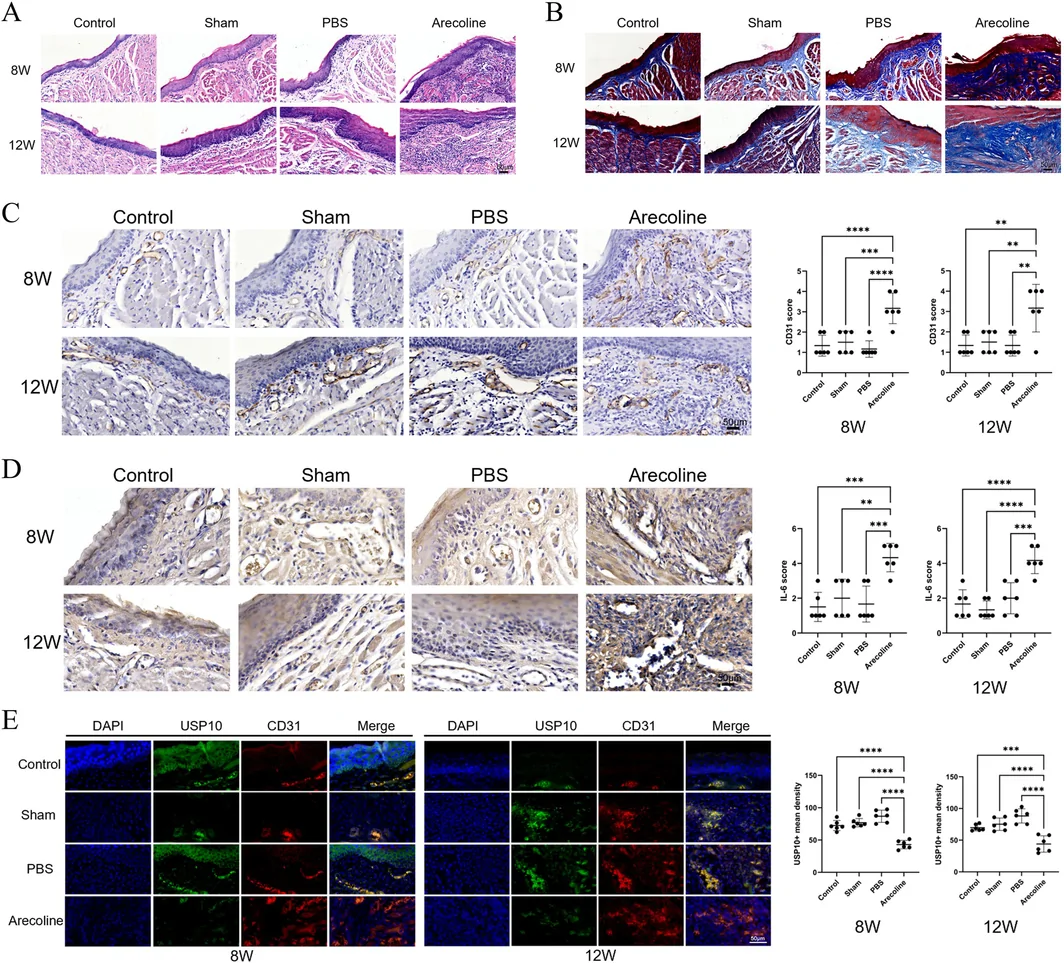
Upregulation of IL‐6 and downregulation of USP10 in mouse OSF model (*n* = 6). (A) HE staining for identifying OSF, scale bar 50 μm. (B) Masson's trichrome staining for identifying OSF, scale bar 50 μm. (C) Representative IHC images and analysis of CD31 and (D) IL‐6 in OSF mouse model, scale bar 50 μm. (E) Immunofluorescence and analysis of USP10 and CD31 in OSF, scale bar 50 μm. **p < 0.01, ***p < 0.001, ****p < 0.0001 by one‐way ANOVA with Dunnett's multiple comparison test.

In order to establish the OSF model, we used a microarray needle with multiple points of simultaneous injection and controlled depth to locally inject arecoline into the left buccal mucosa of mice in the experimental group (Figure S1). HE and MASSON staining revealed that after 8 and 12 weeks of administration, the lamina propria of the buccal mucosa in the arecoline group exhibited noticeable thickening, and the thickness of the subepithelial collagen fibres increased and extension into the submucosa (Figure 2A,B), confirming the successful establishment of the mouse OSF model. Subsequently, IHC was performed to evaluate vascular changes in the mouse OSF model. After 8 and 12 weeks of treatment, the arecoline group exhibited increased vascular density, accompanied by extensive inflammatory cell infiltration and collagen fibre deposition around the vessels (Figure 2C), resembling the pathological features of early stage blood vessels in human OSF.

To investigate the expression of the inflammatory factor IL‐6 and USP10 in the mouse OSF model, IHC and IF were conducted. After 8 and 12 weeks of administration, IL‐6 was highly expressed in inflamed blood vessels, while USP10 expression was significantly attenuated (Figure 2D,E).

### 3.3 USP10 Inhibits Angiogenesis by Modulating Endothelial Cell Proliferation and Migration

**FIGURE 3:**
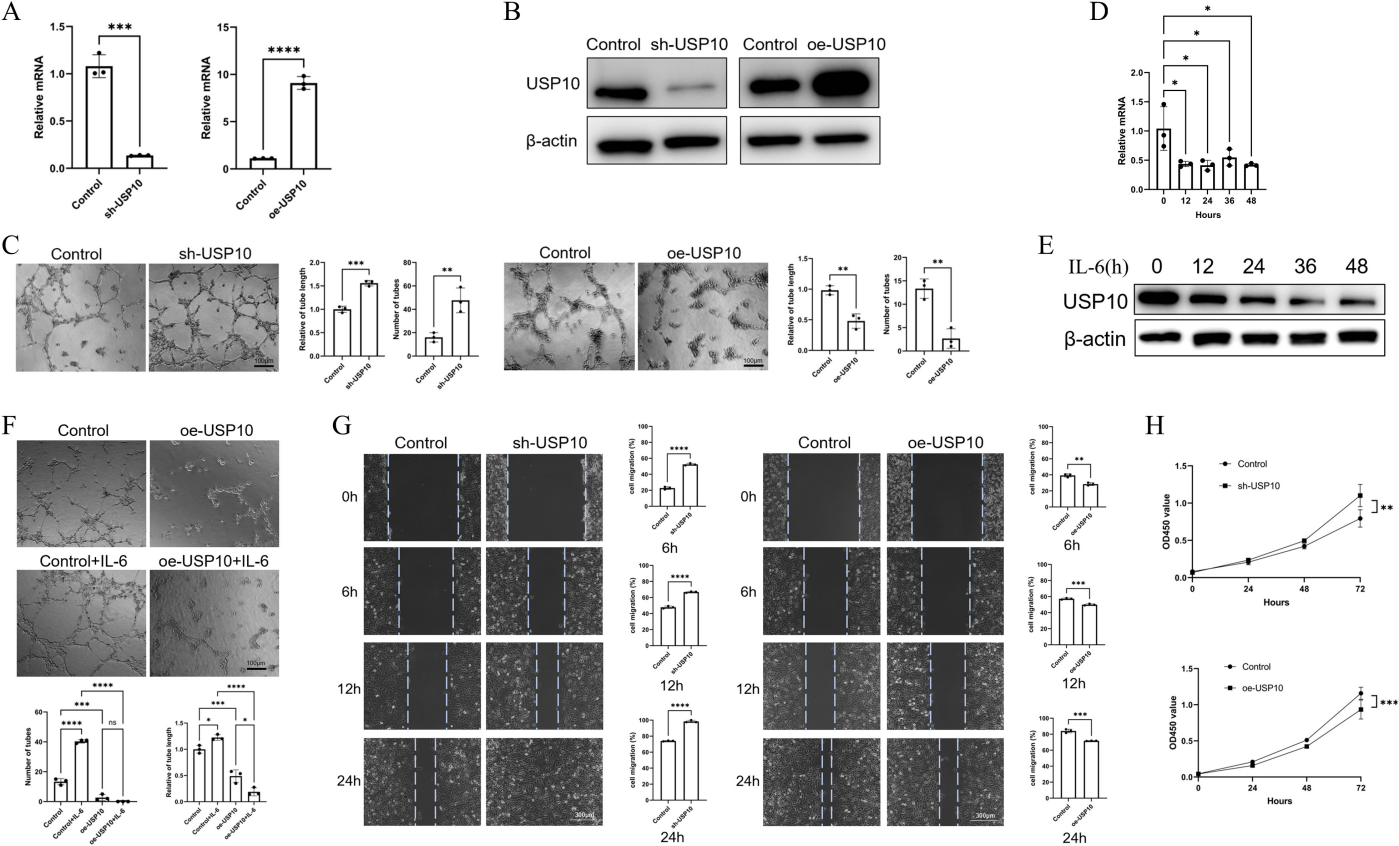
USP10 inhibits angiogenesis, cell migration and cell proliferation in HUVEC. (A) mRNA and (B) protein levels of USP10 in HUVEC after knockdown or overexpression of USP10. (C) Tube formation assay and statistical quantification of HUVEC, scale bar 100 μm. (D) mRNA and (E) protein levels of USP10 in HUVEC after IL‐6 treatment. (F) Tube formation assay and statistical quantification of HUVEC, scale bar 100 μm. (G) Cell migration assay and statistical quantification of HUVEC, scale bar 300 μm. (H) Cell proliferation assay of HUVEC. Data are representative of *n* = 3 independent experiments. *p < 0.05, **p < 0.01, ***p < 0.001, ****p < 0.0001, NS means no significance by unpaired Student's *t* test or one‐way ANOVA with Tukey's multiple comparison test.

To elucidate the biological functions of USP10 in HUVECs, we initially established USP10 knockdown and overexpression cells using specific shRNA transfection, as confirmed by qPCR and WB analyses (Figure 3A,B). To further investigate the role of USP10 in angiogenesis, we conducted tube formation assays, which revealed that USP10 significantly inhibits angiogenesis in HUVECs (Figure 3C).

In addition, to investigate the effect of IL‐6 on the expression level of USP10, we used 50 ng/mL of recombinant human derived IL‐6 protein to treat HUVEC. The results showed that IL‐6 could inhibit the expression of USP10 (Figure 3D,E). Previous studies have shown that IL‐6 can confer angiogenesis promoting properties to microglia [9]. To investigate the impact of IL‐6 on angiogenesis in HUVECs, we further examined changes in tube formation ability following IL‐6 treatment. The results revealed that IL‐6 promoted angiogenesis in HUVECs. To further investigate the effect of targeting USP10 in IL‐6 mediated pathological angiogenesis, we overexpressed USP10 in HUVECs and evaluated the tube formation ability after IL‐6 treatment. The results indicated that overexpression of USP10 effectively reversed IL‐6 mediated pathological angiogenesis (Figure 3F).

Furthermore, we conducted wound healing and cell proliferation assays, which demonstrated that USP10 effectively suppresses both cell migration and proliferation in HUVECs (Figure 3G,H).

### 3.4 USP10 Interacts With and Stabilizes VEGFR1 During Angiogenesis

**FIGURE 4:**
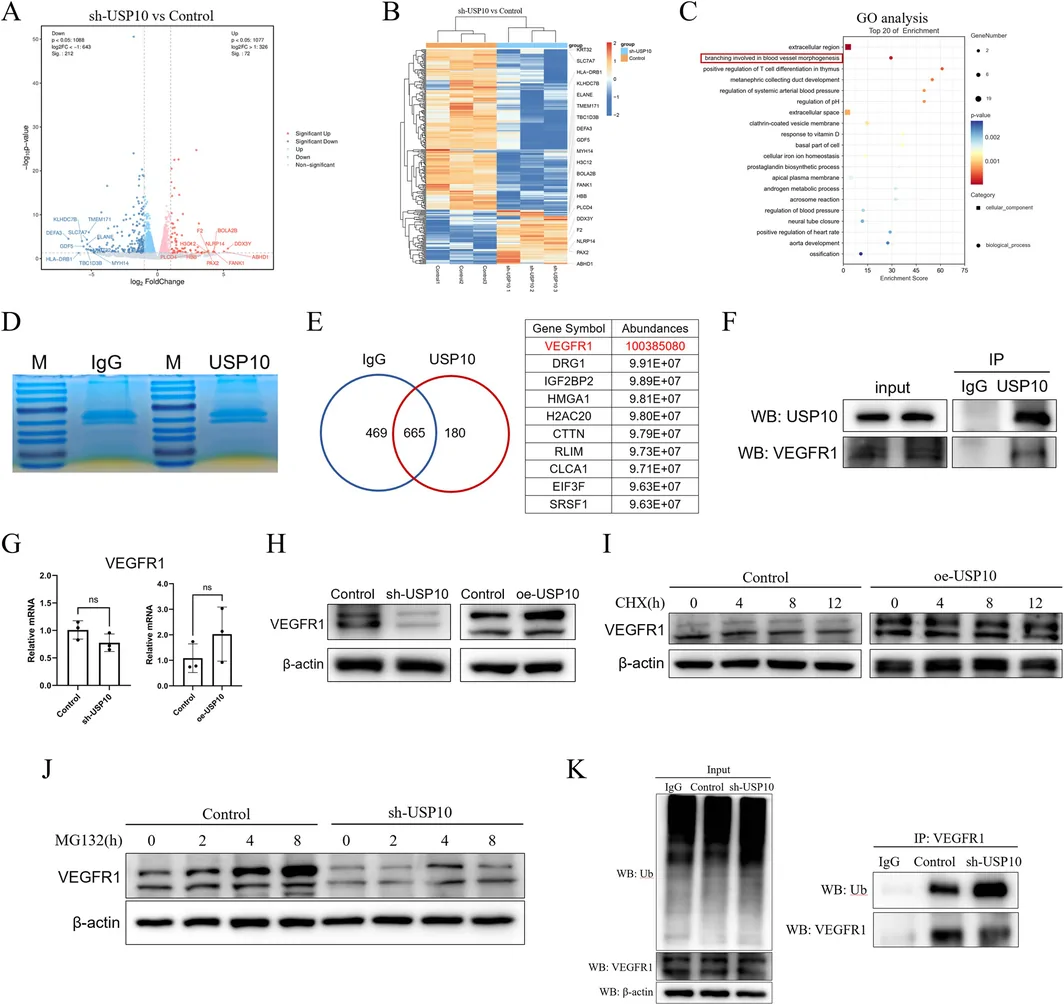
Molecular mechanism of USP10 regulation of angiogenesis. (A) Volcano plot of differential expression genes from RNA‐seq analysis of HUVEC after USP10 knockdown. (B) Heatmap showing high intra‐group reproducibility. (C) GO analysis of the significantly changed pathways identified. (D) The immunoprecipitation of USP10 interacting protein was visualized by Coomassie blue staining. (E) Venn diagram showing the number of proteins specifically interacting with USP10, and ranks the highly abundant proteins. (F) Immunoprecipitation experiments showed that USP10 and VEGFR1 have a direct interaction relationship. (G) mRNA levels or (H) Protein levels of VEGFR1 in HUVEC. (I) CHX (100 μg/mL) or (J) MG132 (10 μM) for the indicated periods of time. (K) The ubiquitylation assay showed the ubiquitination degradation process of the VEGFR1. Data are representative of *n* = 3 independent experiments. NS means no significance by unpaired Student's t test.

We have demonstrated that USP10 inhibits angiogenesis in HUVECs. To elucidate the underlying regulatory mechanisms, we performed RNA‐seq analysis to identify differentially expressed genes (DEGs) in HUVECs following USP10 knockdown. The analysis revealed 284 DEGs, including 72 up‐regulated and 212 down‐regulated genes (Figure 4A). Hierarchical clustering of these DEGs demonstrated high intra‐group reproducibility (Figure 4B). To gain further insights into the functional implications of these DEGs, we conducted Gene Ontology (GO) analysis, with a particular focus on vascular‐related pathways. The results indicated activation of pathways associated with vascular morphogenesis following USP10 knockdown, which aligns with our previous experimental findings (Figure 4C).

To investigate the specific protein that USP10 directly interacts with and their potential impact on angiogenesis, we conducted mass spectrometry analysis. The analysis identified 180 proteins that specifically interact with USP10. Upon ranking these proteins, we observed that USP10 interacts with several proteins in high abundance, among which VEGFR1 has been previously reported to be associated with angiogenesis (Figure 4D,E) [22]. Previous studies have demonstrated that during angiogenic sprouting, endothelial cells migrate and compete for the tip position, which is influenced by the extracellular microenvironment and the expression levels of VEGFR on the cell surface. Specifically, endothelial cells with elevated VEGFR2 and reduced VEGFR1 expression are more likely to attain and maintain the leading position [22, 23]. To validate the mass spectrometry findings, we performed Co‐IP experiments. The results showed that VEGFR1 protein interacted with USP10 protein (Figure 4F). Adenovirus‐mediated knockdown of USP10 resulted in reduced VEGFR1 protein but not its mRNA expression levels in HUVEC. Consistent with these findings, overexpression of USP10 yielded analogous effects. These results collectively suggest that USP10 regulates VEGFR1 expression at the post‐translational level (Figure 4G,H). To investigate the mechanism underlying USP10‐regulated VEGFR1 upregulation, we pretreated HUVECs with cycloheximide (CHX) to inhibit protein synthesis and monitor the degradation of VEGFR1. We found that this treatment had a limited effect on VEGFR1 degradation when USP10 appeared (Figure 4I). Consistent with this result, knockdown of USP10 in HUVECs treated with the proteasome inhibitor MG132 led to a marked reduction in VEGFR1 protein levels (Figure 4J). Collectively, these results indicate that USP10 enhances expression primarily by enhancing VEGFR1 stability. To determine whether USP10 stabilizes VEGFR1 by inhibiting its ubiquitination degradation, we performed a ubiquitination assay. The results demonstrated that VEGFR1 has a ubiquitination degradation process and that USP10 effectively suppresses the ubiquitination level of VEGFR1 (Figure 4K). Based on these experimental results, we conclude that USP10 interacts with VEGFR1, inhibiting its ubiquitination and increasing its expression in HUVECs, thereby suppressing angiogenesis.

## 4 Discussion

In recent years, the prevalence of OSF has been steadily increasing, accompanied by a rising number of patients and a significant escalation in the rate of malignant transformation. A substantial proportion of patients are diagnosed at middle to advanced stages of the disease, which complicates treatment and reduces therapeutic efficacy. Consequently, elucidating the pathogenesis of OSF and developing strategies to control disease progression at an early stage represent critical clinical and scientific challenges.

Fibrosis is a protective response to inflammation and tissue injury, characterized by the infiltration of cells into the injured area and the secretion of excessive extracellular matrix (primarily Type I collagen) to repair and reinforce damaged tissue, thereby accelerating the healing process. However, fibrotic dysregulation caused by repetitive tissue injury results in the persistent activation of matrix secreting fibroblasts and myofibroblasts, ultimately altering tissue architecture and leading to organ dysfunction [24, 25, 26]. Evidence indicates that the progression of organ fibrosis is accompanied by pathological angiogenesis [27, 28, 29]. Under pathological conditions, inflammation and hypoxia act as key drivers of vascularization. This immature angiogenesis occurring in fibrotic organs is ineffective at alleviating tissue hypoxia and may thus exacerbate organ damage and fibrotic progression [30, 31, 32]. Numerous evidence suggests that inhibiting angiogenesis can normalize distorted vasculature in fibrotic livers and attenuate liver fibrosis [33, 34, 35]. In the early stages of OSF, hypoxia and localized inflammation synergistically drive angiogenesis. However, the resulting immature blood vessels fail to alleviate tissue hypoxia and instead exacerbate cytokine production, further stimulating the generation and accumulation of extracellular matrix, thereby accelerating OSF progression [5, 36, 37]. Most studies have shown that OSF is characterized by extensive neovascularization and a pronounced inflammatory response. Microvessel density, vessel lumen diameter, and vessel area are elevated in the early stage of OSF but decline in the middle and late stages. This early‐stage increase in angiogenesis is likely attributed to inflammation and hypoxia triggering angiogenesis [36, 37, 38, 39, 40, 41, 42, 43]. However, Singh M et al. reported that vascular density gradually decreases as OSF progresses, while vessel area and diameter increase in the early stage due to compensatory vascular dilation, but decline significantly in middle to late stages [44]. Consequently, therapeutic strategies for early stage of OSF could involve inhibiting pathological angiogenesis together with controlling inflammation and hypoxia. Despite these findings, the mechanisms underlying angiogenic changes in OSF remain poorly understood, with most studies focusing on vascular alterations in the middle and late stages of the disease [42, 45, 46]. For instance, Wang J et al. reported significant upregulation of Notch in OSF, with Notch inhibition leading to improved ischemic conditions and reduced collagen deposition [45]. Similarly, Yang X et al. demonstrated high expression of THBS1 in OSF, which is associated with inhibition of angiogenesis. Moreover, inhibition of THBS1 can reduce collagen deposition [46]. Lin Y et al. revealed that the PI3K/Akt pathway regulates VEGF expression, causing compensatory vascular hyperplasia in early stage of OSF, while inhibition of this pathway was associated with reduced vascularization in the late stage [42]. In contrast, the present study emphasizes the early stage of OSF, aiming to identify strategies to reverse disease progression at its onset. Consistent with existing literature, we observed increased vascular density and significant inflammatory cell infiltration around blood vessels, surrounded by collagen fibres in early stage of OSF. Additionally, the progression of OSF is associated with elevated expression of inflammatory mediators and their receptors, including IL‐1β, TNF‐α, and IL‐6. Current research on IL‐6 in OSF has primarily focused on its upregulated expression in early stage OSF tissues [10]. However, no studies have explored the relationship between IL‐6 and vascular changes in OSF. In this study, we identified high expression of IL‐6 in inflammatory vessels during the early stages of OSF, suggesting that IL‐6 may contribute to OSF progression by modulating angiogenesis.

Currently, there is a limited number of studies on animal models of OSF both domestically and internationally. Early research by Sirsat et al. attempted to induce OSF lesions by applying capsaicin to the palatal mucosa of rats [47]. However, the pathological manifestations were atypical. Similarly, Chiang et al. developed an OSF model by feeding hamsters fresh betel nut over an 18 month experimental period, but the pathology primarily showed atypical epithelial hyperkeratosis and spinous layer hyperplasia [48]. Sumeth Perera et al. established a mouse OSF model by topically applying betel nut extract to the buccal mucosa for over 1 year, achieving pathological changes similar to human OSF [49]. However, the prolonged induction period led to age‐related changes in the oral mucosa, including collagen fibre cleavage and vitreous changes in the lamina propria, which could confound experimental results. Wen et al. constructed a mouse OSF model using arecoline drinking water, but this method exerted systemic effects on the mice [50]. Zhang et al. developed an OSF model by locally injecting bleomycin into the buccal mucosa of rats [51]. However, bleomycin is not a natural food component, and its fibrotic mechanism differs from that of betel nut. Additionally, bleomycin induces pulmonary fibrosis in rats. In recent years, a more widely adopted method involves the local injection of arecoline into the buccal mucosa of rats or mice [46, 52, 53]. However, conventional injection needles are relatively thick, causing significant damage to the buccal mucosa, and the injection depth is often inconsistent. These limitations were observed in the early stages of our research, resulting in prolonged modelling periods and unstable outcomes. All the aforementioned OSF modelling methods exhibit certain limitations. In this study, we employed a microarray needle to locally inject arecoline into the left buccal mucosa of mice, enabling simultaneous multi‐point injections with controlled depth. Our arecoline induced OSF model simulates the oral environment of betel nut chewers, and the pathological features of the mouse model closely resemble early vascular changes observed in human OSF. In summary, we present a stable and rapidly inducible arecoline‐based mouse model for early OSF.

Vascular morphogenesis encompasses a series of tightly regulated processes, including endothelial cell proliferation, migration, tip and stem cell differentiation, lumen formation, branching, anastomosis, and ultimately the formation of a functional vascular network [54]. This intricate process is governed by multiple signalling pathways, such as the VEGF and NOTCH pathways, which are precisely coordinated in both time and space [22]. During angiogenesis, elevated levels of exogenous pro‐angiogenic factors induce the formation of tip cells. These cells extend numerous filopodia to guide the direction of neovascularization and promote fusion with adjacent capillaries, establishing new vascular connections. Simultaneously, stalk cells behind the tip cells proliferate and form luminal structures, contributing to the expansion of the vascular network [55]. USP10 has been shown to reverse the ubiquitination of Notch1, leading to an accumulation of Notch1 in vascular endothelial cells under both nutrient‐rich and hypoxic conditions, ultimately inhibiting endothelial cell sprouting and vascularization [21, 56]. Consistent with these findings, our study demonstrated that USP10 inhibits angiogenesis, migration, and proliferation in HUVECs and negatively regulates pathways associated with vascular morphogenesis.

During angiogenesis, the VEGF/VEGFR signalling is the principal and most clearly defined pathway. VEGFA and its receptors VEGFR1 and VEGFR2 constitute the core signalling axis that governs the angiogenic response [15, 57, 58]. Notably, VEGFR1 exhibits a higher affinity for VEGF compared to VEGFR2. By competitively binding to VEGF, VEGFR1 inhibits the VEGF/VEGFR2 signalling pathway in endothelial cells, thereby suppressing endothelial cell proliferation, migration, and angiogenesis [14, 22, 59]. Multiple studies have further confirmed the inhibitory effect of VEGFR1 on angiogenesis in both in vitro and in vivo settings [18, 19, 59]. The VEGF family is tightly regulated at transcriptional and translational levels to respond to diverse factors, including hypoxia and metabolic changes. Ubiquitination of VEGFR1 is a crucial post‐translational modification, which can regulate the stability, degradation and signal transduction of the protein, thereby directly affecting angiogenesis [57]. Endothelial cells continuously synthesize VEGFR1, while ubiquitin/proteasome activity maintains its steady‐state levels through degradation. Inhibition of ubiquitin or proteasome activity enhances VEGFR1 expression in endothelial cells [60]. Our findings reveal that the USP10 directly interacts with VEGFR1, inhibiting its ubiquitination and increasing its expression in HUVECs, thereby suppressing angiogenesis. Future studies should focus on identifying the specific interaction sites between USP10 and VEGFR1, which could provide a foundation for the development of small‐molecule drugs targeting this pathway for clinical translation.

Our study has several limitations. First, the sample size of clinical specimens was relatively limited. Future studies should aim to validate the relationship between USP10 expression and microvessel density using a larger cohort of human OSF samples. Second, to the best of our knowledge, no prior studies have investigated the relationship between IL‐6 and USP10. In this study, we demonstrated that USP10 is regulated by IL‐6 and that USP10 overexpression reverses IL‐6‐mediated pathological angiogenesis. However, these findings have not yet been thoroughly validated in an in vivo model. Current functional assays rely exclusively on HUVECs, which may not fully recapitulate the physiological characteristics of oral mucosal microvascular endothelium. Consequently, validation in a disease‐relevant endothelial system is warranted in future. Further research is needed to determine whether targeting USP10 can effectively reverse OSF progression at an early stage in vivo, which would provide valuable insights for clinical translation and identify potential targets for drug development.

**FIGURE 5:**
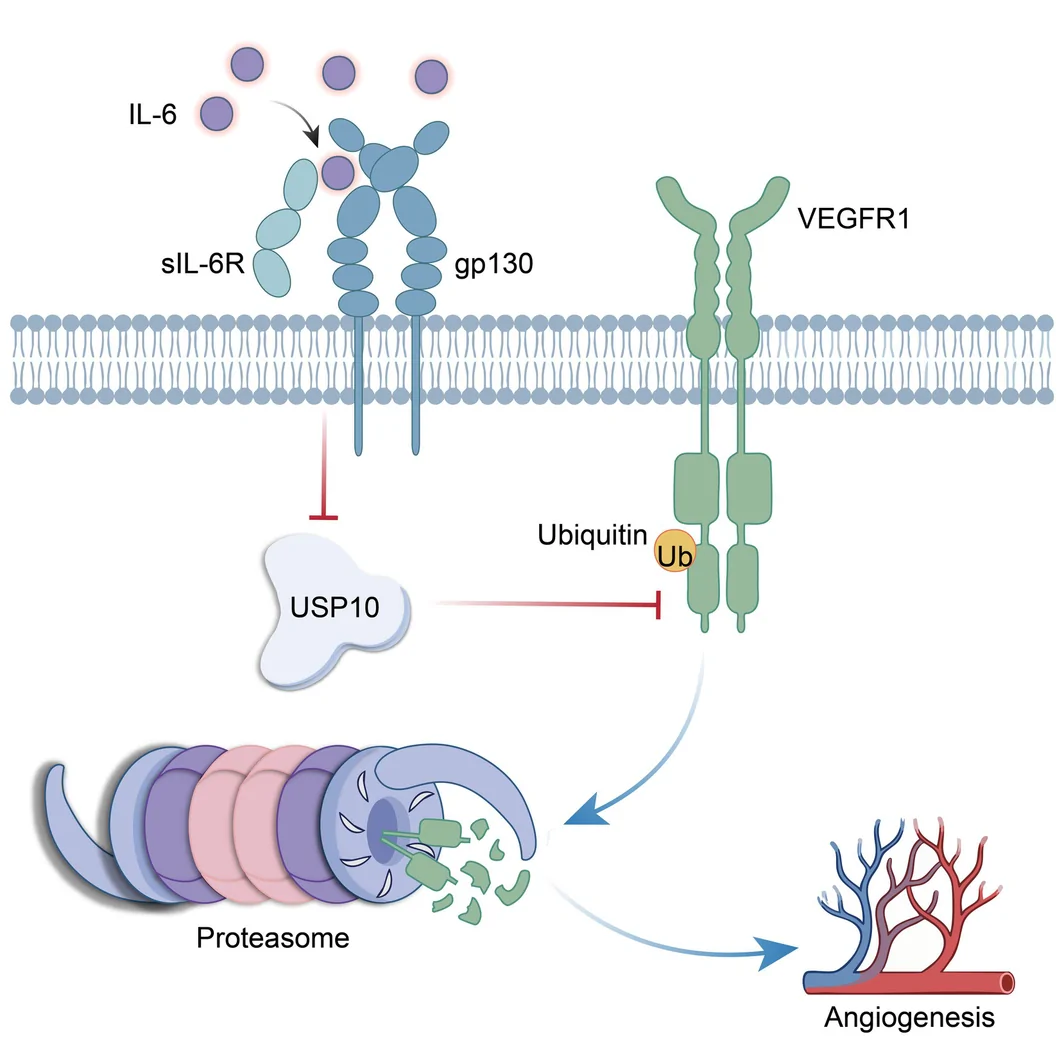
The mechanism of IL‐6‐USP10‐VEGFR1 mediated pathological angiogenesis in OSF.

In conclusion, our findings demonstrate that USP10 may play a role in vascular processes during the early progression of OSF. A possible mechanism is that the expression level of IL‐6 increases during betel nut chewing. Subsequently, IL‐6 first binds to soluble IL‐6 receptor (sIL‐6R), forming a complex and then binds to the adjacent gp130, inducing its dimerization and activation [61]. This activated IL‐6 trans‐signalling pathway promotes inflammatory responses and reduces USP10 expression. Decreased USP10 leads to increased ubiquitination of VEGFR1, thereby targeting it for proteasomal degradation. The reduction in VEGFR1 levels attenuates VEGFA/VEGFR1 signalling, resulting in pathological angiogenesis (Figure 5). Such pathological vascularization further triggers the activation of interstitial cells, including perivascular fibroblasts, and disrupts the balance between collagen synthesis and degradation, ultimately contributing to fibrotic development.

## Author Contributions

**Chunyu Li:** writing – original draft, conceptualization, formal analysis, investigation. **Lingshuang Han:** writing – original draft, formal analysis, investigation. **Zhenhao Wang:** validation. **Yihang Xie:** methodology. **Xiaoping Xu:** data curation, supervision. **Qianming Chen:** conceptualization, funding acquisition, supervision, validation, writing – review and editing. **Liang Xie:** writing – review and editing, conceptualization, funding acquisition, validation, supervision, resources.

## Funding

This work was supported by grants from the Department of Science and Technology of Zhejiang Province (No. 2024C03193), the National Natural Science Foundation of China (81730030, U25A6003), the CAMS Innovation Fund for Medical Sciences (2019‐I2M‐5‐004).

## Ethics Statement

All specimens were obtained from West China Hospital of Stomatology, with the approval of the Ethics Committees of West China Hospital of Stomatology, Sichuan University (WCHSIRB‐D‐2023‐267).

## Consent

The authors have nothing to report.

## Conflicts of Interest

The authors declare no conflicts of interest.

## Supporting information


**Figure S1:** Microarray needle with multiple points of simultaneous injection.

**Table S1:** Clinicopathological features of patients with OSF.

## Data Availability

All data generated of analysed during this study are included in this published article and its Supporting Information. The datasets generated and used in this study are available from the corresponding author on reasonable request.
