# Supplementary material for: IL‐6 Promotes Pathological Angiogenesis in Oral Submucous Fibrosis by Suppressing USP10–VEGFR1 Interaction
Source: J Cell Mol Med. 2026 Aug 19;30(16):e71324. doi: 10.1111/jcmm.71324 (PMC13490721; doi:10.1111/jcmm.71324)
Supplement: Supplementary file 1 [file JCMM-30-e71324-s002.docx]

Table S1 Clinicopathological features of patients with OSF

|  | Cases | Percent (%) |
| --- | --- | --- |
| Total | n=6 |  |
| **Gender** |  |  |
| Male | 4 | 66.67 |
| Female | 2 | 33.33 |
| **Age (median), yr** | 33（20-47） |  |
| **Betel nut chewing** |  |  |
| Current | 1 | 16.67 |
| Former | 5 | 83.33 |
| **Smoking** |  |  |
| Current | 2 | 33.33 |
| Former | 3 | 50 |
| Never | 1 | 16.67 |
| **Histologic type** |  |  |
| Early stage | 6 | 100 |
|  |  |  |
